# Pain Related Cortical Oscillations: Methodological Advances and Potential Applications

**DOI:** 10.3389/fncom.2016.00009

**Published:** 2016-02-04

**Authors:** Weiwei Peng, Dandan Tang

**Affiliations:** ^1^Key Laboratory of Cognition and Personality (Ministry of Education), Faculty of Psychology, Southwest UniversityChongqing, China; ^2^School of Education Science, Zunyi Normal CollegeGuizhou, China

**Keywords:** pain, cortical oscillations, event-related desynchronization (ERD), event-related synchronization (ERS), electroencephalography (EEG)

## Abstract

Alongside the time-locked event-related potentials (ERPs), nociceptive somatosensory inputs can induce modulations of ongoing oscillations, appeared as event-related synchronization or desynchronization (ERS/ERD) in different frequency bands. These ERD/ERS activities are suggested to reflect various aspects of pain perception, including the representation, encoding, assessment, and integration of the nociceptive sensory inputs, as well as behavioral responses to pain, even the precise details of their roles remain unclear. Previous studies investigating the functional relevance of ERD/ERS activities in pain perception were normally done by assessing their latencies, frequencies, magnitudes, and scalp distributions, which would be then correlated with subjective pain perception or stimulus intensity. Nevertheless, these temporal, spectral, and spatial profiles of stimulus induced ERD/ERS could only partly reveal the dynamics of brain oscillatory activities. Indeed, additional parameters, including but not limited to, phase, neural generator, and cross frequency couplings, should be paid attention to comprehensively and systemically evaluate the dynamics of oscillatory activities associated with pain perception and behavior. This would be crucial in exploring the psychophysiological mechanisms of neural oscillation, and in understanding the neural functions of cortical oscillations involved in pain perception and behavior. Notably, some chronic pain (e.g., neurogenic pain and complex regional pain syndrome) patients are often associated with the occurrence of abnormal synchronized oscillatory brain activities, and selectively modulating cortical oscillatory activities has been showed to be a potential therapy strategy to relieve pain with the application of neurostimulation techniques, e.g., repeated transcranial magnetic stimulation (rTMS) and transcranial alternating current stimulation (tACS). Thus, the investigation of the oscillatory activities proceeding from phenomenology to function, opens new perspectives to address questions in human pain psychophysiology and pathophysiology, thereby promoting the establishment of rational therapeutic strategy.

## Introduction

Pain, affecting the well beings of millions of individuals and imposing a severe financial burden upon our societies, is a major public healthcare problem. Pain relief, especially for the patients with pathological chronic pain, still remains a very problematic challenge to the physicians. The progress in understanding of the neural representation of pain in humans is not only important for basic neuroscience research, but also critical to develop effective strategies for the diagnosis and management of the pathological pain conditions. Specifically, this constitutes the understandings of: (1) the physiological mechanisms of the nociceptive system in healthy populations, particularly the cortical processes underlying the perception of pain and (2) the pathophysiological mechanisms of the nociceptive system in chronic pain patients, particularly the peripheral and central mechanisms leading to chronic pain. Thus, for a better understanding of the physiology and pathophysiology of pain in humans, novel approaches should be developed to identify the neural activities related to the processing of noxious inputs in humans, as well as characterize their functional roles in subjective pain perception.

**Figure 1:**
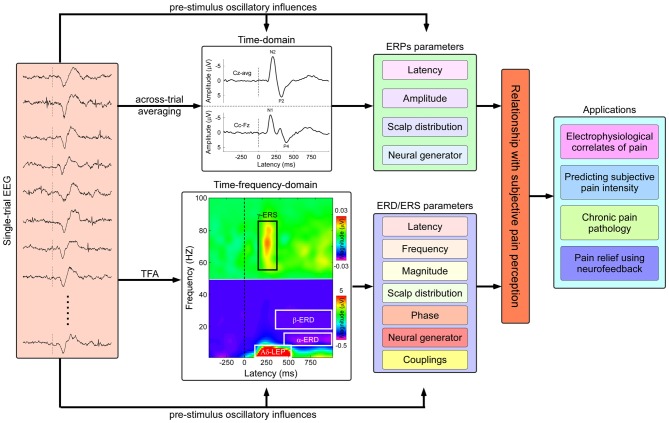
**Flowchart describing the identification of pain related electrophysiological features and applications in both basic and clinical pain study.** Nociceptive somatosensory inputs can elicit transient changes in the ongoing electroencephalography (EEG) activities, including phase-locked event-related potentials (ERPs) and non-phase-locked modulations of ongoing oscillatory activities in multiple frequency bands (appeared as event-related desynchronization or synchronization, ERD or ERS). The phase-locked ERPs activities could be obtained by the classical across-trial averaging process, characterized by their peak latency, amplitude, scalp topography, and neural generators, while the non-phase-locked ERD/ERS activities could be identified using time-frequency analysis (TFA), characterized by several parameters including latency, frequency, magnitude, scalp topography, phase, neural generator, casual information flows, and cross-frequency couplings (CFCs). The assessment of the relationship between human pain perception and electrophysiological responses has the potential applications in both basic and clinical pain study, including: (1) exploring electrophysiological signatures coding subjective pain perception; (2) predicting subjective pain intensity; (3) exploring pathological mechanisms of chronic pain; and (4) relieving pain modulating the cortical oscillatory activities using neurofeedback techniques.

In both physiological (Iannetti et al., 2003) and pathophysiological (Treede, 2003; Treede et al., 2003) studies, laser-evoked potentials (LEPs) have been extensively used to investigate the peripheral and central processing of nociceptive somatosensory inputs, and are currently considered as the best available diagnostic tool to assess the function of nociceptive pathways in patients (Cruccu et al., 2010). The radiant heat pulses that selectively excite nociceptive nerve endings in the epidermis (Bromm et al., 1984), can elicit a number of electrical brain responses, some of which can be detected with the electroencephalography (EEG) recording techniques (Carmon et al., 1976; Mouraux et al., 2003). Note that the EEG response is time-locked if it manifests the same pattern at roughly the same time on each trial after the stimulus onset, and the EEG response is phase-locked if it takes the same phase angle on each trial after the stimulus onset (Mouraux and Iannetti, 2008). The time-locked and phase-locked LEPs could be commonly obtained by an across-trial averaging procedure. Several deflections have been identified in LEPs (Figure 1), including: (1) an early component of a small negative deflection (N1, peaking at approximately 160 ms when stimulating the hand dorsum), with maximal distribution over the central temporal region contralateral to the stimulated side (Valentini et al., 2012); (2) the largest deflection of a negative-positive vertex potential (N2-P2 complex, peaking at approximately 160 and 390 ms when stimulating the hand dorsum), with maximal scalp distribution over the central region (Iannetti et al., 2008); and (3) a late component of a positive deflection (P4, approximately 390 ms when stimulating the hand dorsum), with maximal scalp distribution over the central–parietal region contralateral to the stimulated side (Hu et al., 2014a). As revealed by dipole modelings of scalp, subdural recordings, and direct intracranial recordings (Tarkka and Treede, 1993; Bromm and Chen, 1995; Lenz et al., 2000; Garcia-Larrea et al., 2003; Valentini et al., 2012), LEPs were showed to be generated from a combination of cortical and subcortical structures, including the primary and secondary somatosensory cortex (S1 and S2), insula, and anterior/mid-cingulate cortex (ACC/MCC), as well as parietal operculum. Functionally, recent evidences (Iannetti et al., 2008; Mouraux and Iannetti, 2009) showed that these laser-evoked EEG responses represent an indirect readout of the function of nociceptive system, mainly determined by the saliency of the eliciting nociceptive stimulus, i.e., the ability to capture attention, instead of the specific neural processes underlying pain perception.

Alongside the ERPs, sensory stimuli could also induce transient modulations of the ongoing oscillatory activities in different frequency bands (Pfurtscheller and Lopes da Silva, 1999). Since these oscillatory activities are normally time-locked but not phase-locked to the onset of the stimulus, they would be eliminated by the classical across-trial averaging procedures that are typically used to reveal ERPs (Mouraux and Iannetti, 2008). Alternative signal processing techniques, based on the joint time-frequency decompositions of signals, are often adopted to explore the neurophysiological mechanisms of brain oscillations. These modulations are characterized by either transient enhancement (event-related synchronization, ERS) or transient suppression (event-related desynchronization, ERD) of the oscillation power, usually confined to a specific frequency band (Pfurtscheller and Lopes da Silva, 1999). The functional significance of ERS and ERD differs according to the frequency band within which they occur. For example, ERD in the alpha band (frequencies ranging from 8–13 Hz) has been hypothesized to reflect cortical activation or disinhibition (Pfurtscheller and Lopes da Silva, 1999; Schnitzler et al., 2000; Hu et al., 2013), while ERS in the gamma band (frequencies ranging from 30–100 Hz) has been hypothesized to play a crucial role in cortical integration and perception (Tallon-Baudry and Bertrand, 1999; Gross et al., 2007; Fries, 2009; Hipp et al., 2011).

By performing time–frequency analysis on the EEG signals elicited by nociceptive somatosensory stimuli, several electrophysiological responses (ERPs) related to the activation of nociceptive fibers have been disclosed (Figure 1), including: (1) a suppression of the alpha oscillations, i.e., α-ERD, globally across somatosensory, motor, and visual areas, reflecting a widespread change of cortical function and excitability, and relating to the special alerting function of pain (Mouraux et al., 2003; Ploner et al., 2006b; Hu et al., 2013); (2) a suppression of beta oscillations (~20 Hz in frequency), i.e., β-ERD, predominantly over the contralateral primary motor cortex without an obvious beta oscillation rebound followed (Raij et al., 2004), indicating the prolonged excitations of neurons within motor cortex, which may be associated with the facilitation of the voluntary movements to prevent tissue damage in pain processing; and (3) enhancement of gamma oscillations, i.e., γ-ERS, over contralateral somatosensory cortex, particularly relating to subjective pain intensity (Gross et al., 2007; Zhang et al., 2012; Hu et al., 2014b), and reflecting the internal representations of behaviorally relevant stimuli that should receive enhanced/preferred processing.

These painful stimulus induced ERD/ERS responses, occurring in painful information processing, have been suggested to be associated with the perception of pain (Babiloni et al., 2006; Gross et al., 2007; Zhang et al., 2012) and with endogenous or exogenous attention to the painful stimuli (Mouraux et al., 2003; Hauck et al., 2007; Hu et al., 2013). However, it is still not clear whether these somatic sensory pain-related oscillatory activities are pain-specific opposed to non-painful somatosensory stimuli, or the salience of the stimuli presentation (Iannetti et al., 2008; Mouraux and Iannetti, 2009). Even though, these stimulus induced ERD/ERS activities could indeed provide plentiful information related to brain processing, which is different from those cortical activities reflected by stimulus-evoked ERPs (Mouraux and Iannetti, 2008). Previous studies have indicated that nociceptive somatosensory stimuli induced ERD/ERS activities in multiple frequency bands could reflect various aspects of pain perception (e.g., representation, encoding, assessment, and integration of the nociceptive sensory stimuli, as well as the behavioral responses to pain), even the precise details of their roles remain unclear. Indeed, investigating the cortical oscillatory activities involved in human pain perception and establishing the oscillatory basis of pain opened a new window to study the cortical process underlying pain perception. Thus, in this article, we will: (1) highlight several methodological recommendations on investigating brain oscillations related to pain and (2) summarize the potential applications in both basic and clinical pain study.

## Methodological Recommendations to Extract Pain Related Brain Oscillatory Activities

The transient modulations of cortical oscillatory activities induced by the nociceptive somatosensory stimuli are normally characterized by their peak frequency, latency, magnitude, and topography distribution, relative to the baseline period (using subtraction or percentage approach). Nevertheless, the traditionally temporal, spectral, and spatial profiles can only partly reveal the dynamics of brain oscillatory activities. Investigating novel parameters comprehensively characterizing brain oscillations could help explore the psychophysiological mechanisms of neural oscillations, as well as the neural functions of cortical oscillations involved in sensory perception and behavior. In addition, pre-stimulus ongoing EEG oscillation could influence both post-stimulus electrophysiological activities and sensory perception (Thut et al., 2006; Romei et al., 2008; Fellinger et al., 2011; Lange et al., 2012; Tu et al., 2016), suggesting the importance of dissecting the contributions of pre- and post-stimulus oscillation to the variabilities of painful stimulus induced ERD/ERS activities. Based on these understandings, from the methodological aspect, we encourage the researchers of pain field to: (1) utilize novel parameters to comprehensively characterize pain related oscillations and (2) dissect the contributions of pre- and post-stimulus oscillations, when they are investigating the dynamics of brain oscillatory activities associated with pain perception and behavior.

### Utilization of Novel Parameters to Comprehensively Characterize Pain Related Oscillations

Apart from the appearing frequency, latency, magnitude, and scalp topography, several other parameters, including but not limited to, the phase, neural generator, and cross-frequency coupling (CFC) of pain related oscillations, should also be further investigated, for a comprehensive and systemic understanding of the brain oscillations associated with pain.

#### Phase

Much of the research on oscillations in human EEG has focused on the dynamics of oscillations magnitudes. Nevertheless, the phase of the oscillatory activities at a given frequency band reflects cyclic fluctuations of a network’s excitability and varies on a much faster timescale than the sluggish amplitude fluctuations at the same frequency band (Buzsáki and Draguhn, 2004; Lakatos et al., 2005; Rajkai et al., 2008), the phase of the oscillations may provide deep insights into the fine-grained neural mechanisms underlying sensory perception (Buzsáki and Draguhn, 2004; Busch et al., 2009). Indeed, it is suggested that phase synchronization between alpha oscillations in different brain areas allows for an effective network communication and information transmission regulation (von Stein and Sarnthein, 2000; Palva and Palva, 2011; Saalmann et al., 2012).

A growing body of studies on EEG oscillations have shown that the phase of ongoing theta and alpha frequency oscillations prior to the onset of stimuli could influence both the subsequent ERPs (e.g., Haig and Gordon, 1998; Kruglikov and Schiff, 2003; Gruber et al., 2005; Fellinger et al., 2011) and sensory stimulus perception (Busch et al., 2009; Mathewson et al., 2009). As shown in the target auditory oddball data, the amplitude of ERPs (e.g., N100 amplitude) as well as the reaction times (RTs) were both significantly modulated by the phase synchronization of the alpha oscillation that was evaluated by the angular variance of the oscillation (Haig and Gordon, 1998). Using identical visual stimuli at the individual detection threshold (Busch et al., 2009), the phase of ongoing oscillation (in theta and alpha frequency bands) accounted for about 16% of variabilities of visual detection performance (hits or misses) and allowed the prediction of sensory performance on the single-trial level. In other words, the phase of ongoing oscillations reflects the cortical processing of threshold visual stimuli, thus providing a direct link between phase of oscillations and sensory perception and behavior.

These evidences of a relationship between spontaneous oscillation phase and the amplitude of subsequent ERPs, manual responses, and sensory perception, are in line with the cellular level concept that the neuronal oscillations reflect the cyclic variations of neuronal excitability (Buzsáki and Draguhn, 2004; Rajkai et al., 2008). Even the dynamics of phase information in cortical oscillatory activities have been shown to be functionally relevant in stimulus processing and perception of auditory, visual, and even somatosensory modalities, the modulations of pain elicited ERPs as well as pain perception and behavior by the phase of the oscillatory activities, still remain unclear. It therefore needs further investigation, which could broaden the understanding regarding how the ongoing oscillations shape our sensory painful perception.

#### Neural Generators

The spatial characteristics of stimulus induced ERD/ERS activities could be based on their scalp topographies, but the effects of active references in EEG recordings could not be denied. Whether the reference problems in assessing ERD/ERS oscillatory activities could be reduced by approximately standardizing the reference of scalp EEG recordings to a point at infinity, which was ever proposed in assessing evoked potentials by Yao (2001), should be further investigated. Nevertheless, the fact that the equivalent sources of evoked potentials and oscillatory activities are actually independent from the choice of a particular reference, suggests the importance of identifying neural generators of stimulus-induced ERD/ERS activities. With accumulating evidence showing the functions of the oscillatory brain activities in various aspects of pain perception (Mouraux et al., 2003; Ploner et al., 2006a, b; Gross et al., 2007; Zhang et al., 2012; Hu et al., 2013), identifying sources of oscillatory activities is an essential step to directly determine the relation of EEG oscillations to brain function and sensory process, thus revealing how the different cortical areas function as a network involved in human pain perception. For example, alpha oscillations close to the occipito-parietal midline is closely linked to coherent objects (Vanni et al., 1997), suggesting that the function of oscillatory activity in occipitoparietal visual areas in modulating visual shape processing. However, until now, identifying the sources of oscillations in human brain is still a challenging problem due to the low spatial resolution of EEG/MEG recording techniques.

Source localization techniques have been proposed to identify the responsible neural generators (Pascual-Marqui et al., 1994; Cheyne et al., 2003; Hoechstetter et al., 2004; Jurkiewicz et al., 2006; Doesburg et al., 2009), e.g., dipole and distributed source modelings, as well as beamformer technique, and have been adopted in localizing the neural generators of pain related oscillations (Raij et al., 2004; Ploner et al., 2006a, b; Gross et al., 2007; Peng et al., 2012). Gross et al. (2007) computed the painful stimuli induced high-frequency oscillations in the electrical activity of the human S1 using a linearly constrained minimum variance spatial filtering approach, and then the relationships between stimulus induced gamma ERS and objective stimulus intensity as well as subjective pain intensity were established on the source level, making it possible to evaluate the functional relevance of gamma oscillations in pain perception more directly. However, these source localization models are typically ill-posed inverse problems since infinite number of sources could explain a given scalp topography and additional information as constraints is needed to obtain a unique solution. For example, the beamformer source localization technique, which uses an adaptive spatial filter to estimate the activity everywhere in the brain (Gaetz and Cheyne, 2003; Cheyne et al., 2003), is based on minimizing the source power (or variance) at a given location, and assumes that sources in different parts of the brain are not temporally correlated, which does not make sense physiologically sometimes.

Alternative approaches based on the simultaneous recordings of functional magnetic resonance imaging (fMRI) and EEG (Laufs et al., 2003a; Lei et al., 2011; Dong et al., 2014) have also been proposed to explore the neural sources of EEG oscillations by identifying fMRI blood oxygenation level-dependent (BOLD) signal changes related to spontaneous EEG power fluctuations. Even it combines the high spatial resolution in fMRI and high temporal resolution in EEG, such a method of correlating continuously band-specific EEG power with fMRI-BOLD signal changes, is actually an indirect way to identify source of oscillations. Indeed, monitoring the large-scale neuronal firing patterns and the generated local field potentials (LFPs) in animal models (e.g., behaving rodents) serves a direct and effective way to investigate the generators of these various oscillations as well as their spatial and temporal relationships.

#### CFC

As a statistical relationship between oscillatory activities in two different frequency bands, CFCs (may be appeared as phase-to-phase, phase-to-power, or power-to-power couplings) have been proposed to reflect the coordination of neural dynamics across temporal and spatial scales (Canolty and Knight, 2010; Canolty et al., 2006), and have been observed in many species and brain regions. As revealed by the LFPs on monkeys, the phase of low-frequency oscillations was shown to modulate the amplitude of gamma oscillations (Wang et al., 2012), and such CFC was suggest to integrate long-range neural interactions mediated by low-frequency rhythms (e.g., theta/alpha) with local computations mediated by high frequencies (i.e., gamma). Importantly, the abnormal CFC is linked to several cognitive processes and disease states (Schlee et al., 2009; López-Azcárate et al., 2010; Miskovic et al., 2011; de Hemptinne et al., 2013). Couplings between β-phase (13–30 Hz) and γ-amplitude (50–200 Hz) in primary motor cortex showed to be exaggerated for Parkinson patients compared with healthy subjects without motor disorders, and such excessive coupling could be reduced by therapeutic subthalamic nucleus stimulation (de Hemptinne et al., 2013), suggesting the dysfunction of CFC in disease states.

With the evidences showing: (1) the potential relevance of CFC for understanding psychophysiological and pathological brain functions (Canolty et al., 2006; Schlee et al., 2009; Canolty and Knight, 2010; López-Azcárate et al., 2010; Miskovic et al., 2011; de Hemptinne et al., 2013) and (2) nociceptive somatosensory stimuli induced modulations of oscillations in multiple frequency bands (Schulz et al., 2011; Zhang et al., 2012; Hu et al., 2015), we believe that the oscillatory activities in different frequency bands are functioning interactively within the cortical network, and CFCs involved in pain could provide complemented information for the establishment of the cortical oscillatory bases of pain perception. However, it should be noted that the couplings measured anywhere in the brain can be potentially explained by the influence of external sensory inputs or internal cognitive events, on the phase and amplitude of the oscillations, rather than reflecting the actual modulations in different frequency bands. For example, the coupling of theta phase and gamma power observed in rodents (Wang et al., 2011), which was interpreted as a reflection of the storage and processing of nociceptive information, actually can be explained by the common effects of the nociceptive sensory inputs on both theta phase and gamma power, instead of the actual CFC. Therefore, whether the observed correlation between two bands (e.g., phase-amplitude) is due to the common drive, e.g., generated by external or internal input, or whether the correlation is due to a causal interaction between rhythms should be distinguished in the future study.

### Dissection of Pre- and Post-Stimulus Oscillations

The traditional approach to estimate ERD/ERS activities relies on time-frequency decomposition methods to transform the single-trial electrocortical signals into time-frequency distributions (TFDs), and then the resulting TFDs are typically expressed as a percentage change relative to pre-stimulus EEG power to highlight the stimulus-induced changes in power within specific frequency bands (Ploner et al., 2006b; Iannetti et al., 2008; Hu et al., 2013). However, a recent study (Hu et al., 2014b) demonstrated that such baseline percentage approach would introduce a significant bias in estimating ERD/ERS magnitudes, i.e., resulting in an overestimation of ERS and underestimation of ERD, and pointed out that such bias could be avoided using a single-trial baseline subtraction approach.

Importantly, the pre-stimulus oscillatory activities in different frequency bands, reflecting the dynamics of brain states, can influence both the post-stimulus ERPs and sensory perception. For example, the pre-stimulus α-power could significantly modulate the nociceptive-induced α-ERD magnitude (Hu et al., 2013), by showing the nociceptive-induced α-ERD magnitude was significantly more dependent on the pre-stimulus than on the post-stimulus α-power. A more recent study (Tu et al., 2016) showed that the pre-stimulus EEG oscillations in both alpha and gamma frequency bands could significantly modulate the subjective perception of painful stimuli, and importantly, the pre-stimulus alpha and gamma oscillatory activities could provide distinctive information in predicting subjective pain perception. Nevertheless, the single-trial baseline correction approaches (both percentage and subtraction methods) would confuse the contribution of pre- and post-stimulus EEG power, since the baseline corrected ERD/ERS activities reflect the mixed variabilities of changes in the state of the system (reflected as the pre-stimulus oscillations in different frequency bands; Laufs et al., 2003b; Del Percio et al., 2006; Hu et al., 2013) and changes induced by the stimulus and task (reflected as the post-stimulus oscillations).

Thus, it is crucial to dissect the contributions of pre- and post-stimulus power to the variability of ERD/ERS, which reflect different psychophysiological mechanisms. It is proposed to dissect and quantify the relationship between behavioral variables (e.g., RTs and subjective pain intensity) and pre- and post-stimulus EEG activities, e.g., based on a multivariate linear regression model with the combination of partial least square (PLS) regression (Hu et al., 2014b), thus allowing for a full exploration of electrocortical oscillations involved in pain perception.

## Potential Applications in Basic and Clinical Pain Studies

By comprehensively investigating neural oscillatory activities relating to the nociceptive sensory inputs (both transient and tonic stimuli) on healthy subjects, it is likely to establish an oscillatory basis of human pain perception and identify how a network of cortical areas involves in human pain experience. The identification of electrophysiological parameters or signatures encoding how the cortex processes the nociceptive inputs and how the experience of pain may emerge from this complex processing, could indeed open a window to study the cortical process underlying pain function as well as the physiology mechanism of nociceptive systems in humans. In clinical practice, this understanding also would make it possible to predict/measure subjective pain intensity objectively, and definitely help (1) explore the pathological mechanisms of chronic pain and (2) achieve pain relief by modulation the oscillatory activities using neurofeedback techniques, with the investigation of cortical oscillatory activities on chronic pain patients.

### Identifying the Electrophysiological Signatures of Pain Perception

In the last decades, a large number of EEG/MEG studies (Gross et al., 2007; Iannetti et al., 2008; Schulz et al., 2011; Zhang et al., 2012; Hu et al., 2013, 2014a) have extensively investigated the neural activities in response to the various kinds of nociceptive stimuli, with focusing specifically on temporal aspects of nociceptive processing. LEPs have been used extensively in the past decades for a progress in the understanding of the cortical processes underlying pain perception, with the assumption that they reflect, at least partly, neural activities specifically involved in processing nociceptive somatosensory inputs. However, Mouraux and Iannetti (2009) demonstrated that nociceptive laser-evoked brain potentials do not reflect nociceptive-specific neural activity by showing: (1) LEPs could be entirely explained by a combination of multimodal neural activities and somatosensory-specific neural activities and (2) the magnitudes of the multimodal activities were significantly correlated with subjective ratings of saliency regardless the sensory modalities.

Nevertheless, with recent evidence showed that: (1) pain induced gamma oscillations over S1 covaried with objective stimulus intensity as well as subjective pain intensity (Gross et al., 2007); (2) the magnitudes of laser induced gamma band oscillations could always predict the subjective pain intensity regardless of the stimulus repetition when applying trains of three laser stimuli with constant 1 s interval (Zhang et al., 2012); and (3) tonic heat pain induced gamma oscillations could significantly predict subjective pain intensity (Peng et al., 2014; Schulz et al., 2015), we speculate that the gamma oscillation may be a candidate of the electrophysiological signatures reflecting nociceptive specific neural activities, even further investigation should be done.

### Predicting Subjective Pain Intensity

Even pain is a subjective first-person experience, and self-report is considered as the golden standard for the evaluation of pain intensity in clinical situations (Cruccu et al., 2010), self-reports of pain intensity are not available in some vulnerable populations which may lead to inadequate or suboptimal treatment of pain. An objective measurement of pain intensity that can complement self-reports, e.g., to monitor the effect of analgesic drug or the recovery of nociceptive system for non-communicative patients, is in demanding in clinical practice. Even it would be optimal to use pain-specific electrophysiological signatures in predicting subjective pain intensity, using the electrophysiological features that are pain-related but not directly specific to pain processing, could also achieve a relatively high accuracy. For example, for an objective evaluation of pain intensity, Huang et al. (2013) used the evoked potentials information (N2 and P2 latencies and amplitudes) of single-trial LEPs, which are considered to mainly reflect attention capture and arousal to the painful stimuli (Iannetti et al., 2008; Mouraux and Iannetti, 2009), with prediction accuracy of ~86.3% and ~80.3% at within-individual and cross-individual level respectively.

Considering (1) the close association between time-frequency oscillatory features (e.g., gamma ERS) with subjective pain intensity (Gross et al., 2007; Zhang et al., 2012); (2) the oscillatory features could provide complementary information of cortical processing that is different from those reflected by evoked potentials (Mouraux and Iannetti, 2008); and (3) the fluctuations of pre-stimulus oscillations could influence and modulate the subsequent sensory perception (Mathewson et al., 2009; Tu et al., 2016), we propose that the prediction of subjective pain intensity is promising to obtain a better performance with the combination information of stimulus-evoked ERPs, stimulus-induced ERD/ERS, and pre-stimulus oscillation in different frequency bands.

### Investigating the Pathological Mechanisms of Chronic Pain: Abnormal Oscillatory Activities in Chronic Pain Patients

Clinical studies have revealed that some chronic pain patients are associated with the occurrence of abnormal cortical oscillatory activities (Sarnthein et al., 2006; Drewes et al., 2008; Sarnthein and Jeanmonod, 2008; Schlee et al., 2009; Walton et al., 2010). By comparing power spectra of the resting EEG of neurogenic pain patients and healthy controls, the patient group exhibited higher resting-EEG power over the frequency range of 2–25 Hz, and the maximal difference appeared in theta frequency band in all electrodes (Sarnthein et al., 2006). Importantly, the excessive theta power gradually decreased and approached normal values after thalamic surgery, suggesting that both EEG and neurogenic pain may be determined by tightly coupled thalamocortical loops (Sarnthein et al., 2006). In addition, the patients with visceral (Drewes et al., 2008) and somatic pain syndromes such as complex regional pain syndrome and neurogenic pain (Sarnthein and Jeanmonod, 2008; Walton et al., 2010) also showed higher baseline levels of delta and/or theta EEG oscillations compared with the healthy controls, localized to the somatosensory cortex corresponding to the pain localization, and to orbitofrontal-temporal cortices related to the affective pain perception. Hepatic encephalopathy patients showed a decreased peak frequency of alpha activity and a delayed alpha rebound in painful stimulus processing over the somatosensory cortex, compared with healthy controls (May et al., 2014). The alternations of the oscillatory activities in chronic pain patients may reflect a dysfunctioned local communication or long-range communication between the functionally specialized assemblies formed by a huge number of neurons in the human brain (Schnitzler and Gross, 2005). Studying the abnormal oscillations in chronic pain patients, could provide insights about the pathological mechanisms underlying chronic pain situations, thus at last leading to a rational basis for the management of pain.

### Relieving Pain by Modulating Cortical Oscillatory Activities using Neurofeedback Techniques

With the evidences showing the association between the ongoing oscillatory activities and subsequent sensory perception and behaviors (Rahn and Başar, 1993a,b; Babiloni et al., 2008; Romei et al., 2008; Lange et al., 2012; Tu et al., 2016), the application of neurostimulation techniques outside the skull, such as repetitive transcranial magnetic stimulation (rTMS) and transcranial alternating current stimulation (tACS) that could selectively modulate the oscillatory activities at specific brain areas (e.g., sensorimotor cortex), is promising to relieve pain (Klein et al., 2015). Using these online stimulation techniques could not only reveal the causal roles of the oscillatory brain activities and subjective pain perception, but also may be considered as effective strategies for clinical pain relief.

Indeed, with the delivery of 20 Hz rTMS over S2, patients with chronic visceral pain exhibited significant analgesic effects (Fregni et al., 2005). In addition, subthreshold motor cortex rTMS at 10 Hz to the chronic neuropathic pain patients, could significantly reduce pain intensity and thermal sensory thresholds in the painful zone, and the pain relief showed to be correlated with the improvement of warmth sensory thresholds (Lefaucheur et al., 2008). They interpret the action of rTMS to patients with chronic pain could induce changes of cortical excitability, thus for a restoration of defective intracortical GABAergic inhibitory processes and the normalization of neuronal activity in thermal sensory relays, since chronic neuropathic pain was associated with the motor cortex disinhibition, which may be related to the impairment of GABAergic neurotransmission responsive to some aspects of pain symptom or to the underlying sensory or motor disturbance. In addition, by testing the effectiveness of tACS over S1 at a wide frequency band (ranging from 2–70 Hz), the tACS over S1 could elicit tactile sensation in a frequency-dependent manner (Feurra et al., 2011), with obvious effects at stimulus frequency within both alpha (10–14 Hz) and high gamma (52–70 Hz) ranges, indicating that online stimulation techniques could be used to reveal the causal roles of the brain oscillations.

## Summary

Besides ERPs, the nociceptive somatosensory inputs could also induce modulations of cortical oscillations, appeared as ERD or ERS in different frequency bands. These ERD/ERS activities are suggested to be involved in different aspects of pain perception (e.g., sensory perception and behavior), even though the details of their functional roles remain unclear. From a methodological perspective, apart from the temporal, spectral, and spatial profiles of the oscillatory activities, it is instructive to adopt novel parameters (e.g., phase, neural generator, and CFC) to comprehensively evaluate the dynamics of cortical oscillations, thus allowing a full exploration of the neuronal oscillations involved in pain perception. Identifying pain related oscillatory activities and establishing an oscillatory basis of pain perception, could lead new insights into the physiological mechanisms of nociceptive systems in humans. In clinical practice, this also offers exciting prospects for the investigation of pathological mechanisms of chronic pain, thus promoting the development of rational therapeutic strategy.

## Author Contributions

WWP wrote and revised the manuscript. DDT revised the manuscript.

## Funding

WWP is supported by the National Natural Science Foundation of China (31500921).

## Conflict of Interest Statement

The authors declare that the research was conducted in the absence of any commercial or financial relationships that could be construed as a potential conflict of interest.
